# How Can Quantitative Analysis Be Used to Improve Occupational Health without Reinforcing Social Inequalities? An Examination of Statistical Methods

**DOI:** 10.3390/ijerph20010019

**Published:** 2022-12-20

**Authors:** Valérie Lederer, Karen Messing, Hélène Sultan-Taïeb

**Affiliations:** 1 Department of Industrial Relations, Université du Québec en Outaouais, Gatineau, QC J8X 3X7, Canada; 2 Department of Biological Sciences, Université du Québec à Montréal, Montreal, QC H3C 3P8, Canada; 3 Department of Organization and Human Resources, School of Management (ESG-UQAM), Université du Québec à Montréal, Montreal, QC H3C 3P8, Canada

**Keywords:** sex, gender, occupational health, epidemiology, statistical methods, intersectional analysis, quantitative analysis, health equity, race, ethnicity

## Abstract

Taking account of sex and gender in occupational health studies poses statistical challenges. Other sociodemographic variables, such as racialization, class, and age, also affect the relations between workplace exposures and health and interact with sex and gender. Our objective was to perform a critical review of conventional and emerging statistical tools, examining whether each analysis takes account of sociodemographic variables (1) in a way that contributes to identification of critical occupational determinants of health (2) while taking account of relevant population characteristics to reflect intersectional approaches to health and (3) using sample sizes and population characteristics available to researchers. A two-step search was conducted: (1) a scientific watch concerning the statistical tools most commonly used in occupational health over the past 20 years; (2) a screening of the 1980–2022 literature with a focus on emerging tools. Our examination shows that regressions with adjustment for confounders and stratification fail to reveal the sociodemographic mechanisms that interact with occupational health problems, endangering the identification of occupational risks. Multilevel (notably MAIHDA) analyses, decision tree, cluster, and latent analyses are useful methods to consider when seeking to orientate prevention. Researchers should consider methods that adequately reveal the mechanisms connecting sociodemographic variables and occupational health outcomes.

## 1. Introduction: Analyzing Sub-Populations to Advance Occupational Health and Equity

Originally excluded [1,2,3] or studied inappropriately [4,5,6,7,8], working women are increasingly included in study populations, and their exposures and occupational health problems are being recognized. However, taking account of sex and gender in occupational health studies poses methodological questions, because both sex and gender can contribute to differences (for example) in job titles, task assignments, work activity, exposures to physical and psychosocial occupational hazards, reactions, and return to work after an occupational illness, all factors that contribute to health outcomes [9,10,11]. 

Sex (biological differences between women and men) and gender (socially defined behaviours and roles associated with being a woman or a man) are two population-level characteristics that can interact with exposures to affect occupational health. Sex is relevant to exposures and outcomes, from the fit of personal protective equipment to effects of environmental toxins. Additionally, sex determines physiological functions that may be affected by occupational exposures, such as spermatogenesis, menstruation, and pregnancy. Gender is linked to occupational health through its relation to job segregation, both vertical (hierarchical position) and horizontal (profession) [12]. At the workplace level, gender can impact task assignments, work methods, and work activity [13], as well as the effects of work schedules [14], work accidents and occupational illnesses on recovery, return to work and work disability compensation [10,15,16]. Since sex and gender can interact to produce workplace exposures and effects, we will use the expression sex/gender to refer to sex, gender, and sex/gender interactions.

Other broad population-level factors such as age, ethnoracial identification, and social class also affect the relation between exposures and effects and provide challenges to occupational health intervention. Therefore, health protection requires attention not only to individual-level risk factors, but also to population-level or “systemic” risk factors, such as discrimination, income insufficiency, and lack of information on population-specific risk factors. With racialized populations, for example, such effect modifiers can include discrimination at work, resulting in more dangerous task assignments or design failure resulting in badly-fitting personal protective equipment [17,18,19,20].

Rose has pointed out the failure of case-control and cohort studies to detect health risks that are common to a whole population [17], later called “Type III errors” by Schwarz and Carpenter [21]. Even where individual-level exposures and vulnerabilities are similar, populations or sub-populations may show great differences in incidence or prevalence of illness. Identifying how such higher-level inequalities operate in workplaces may, in fact, lead to more effective interventions at the workplace level, as well as to improvements in broader policies, laws, and social practices. Indeed, studying sub-populations in occupational health is a prerequisite for orienting interventions aimed at improving equity in the workplace. However, taking into account such differences can be particularly challenging in quantitative designs [5,22,23,24].

Scholars have discussed the need to incorporate different sources of population diversity in health analyses and to guarantee visibility to communities and/or minority groups. Introduced by Kimberlé Crenshaw, the notion of *intersectionality* emerged from U.S. black feminist activism and academic studies, and expresses how social categorizations, such as ethnoracial identification, class, and gender blend to create unique systems of oppression [25]. Originally, this framework was implemented in occupational health research primarily through qualitative methodologies. A complementary notion, *embodiment*, derives from social epidemiology and ecosocial theory. It refers to the processes and pathways through which material inequities, social conditions, power imbalances, and restrained life opportunities affect physiologic functioning [26]. The two approaches converge toward the idea that people integrate various social identities and positions, and that these are reflected in their health. These concepts can also inspire quantitative researchers to consider experiences, exposures, and effects in subgroups that might otherwise go undetected.

In response to calls for more sensitivity to intersectional approaches, researchers have developed new approaches and guidelines for conducting quantitative intersectional research in epidemiology, biomedicine [18,20,27], political science [28], and psychology [29,30].

Our objective was to perform a critical review of conventional, as well as emerging statistical tools, with regard to three analytical criteria: whether the analysis takes account of sociodemographic variables (1) in a way that contributes to identification of critical occupational determinants of health (2) while taking account of relevant population characteristics so as to reflect intersectional approaches to population health, with a particular attention to sex/gender and (3) using sample sizes and population characteristics available to researchers. To our knowledge, such a methodological analysis of available statistical tools in an intersectional perspective applied to occupational health has not previously been provided.

## 2. Methods

We performed a critical review based on the typology of reviews by Grant and Booth [31]. The objective was not to produce a comprehensive synthesis of all available studies in the literature, but to identify the contribution of the most commonly used statistical tools, as well as innovative and alternative tools to the analysis of sociodemographic mechanisms in occupational health. As emphasized by Grant and Booth [31] critical reviews are usually not based on systematic searches, since they do not aim to produce an exhaustive synthesis of research evidence. They are rather focused on identifying critical conceptual contributions and innovations. Such reviews are intended to constitute a starting point for further evaluation. As recommended by Pope et al. [32], we have used a narrative synthesis to map the statistical tools we found according to our three analytical criteria.

### 2.1. Literature Search Strategy

In the first step, we included the statistical analyses most often used in occupational health studies, hereafter called “conventional methods”. These tools were identified by Karran et al. [33] and in biostatistics textbooks [34,35] as widely used in public health and included standard and multilevel regressions, with adjustment, stratification, and/or interaction testing strategies. For this first step, we searched our own databases covering more than 20 years of interest in and research experience with occupational health research, sex/gender, intersectionality, and equity [5,10,36]. We had collected studies, reviews, and methodological articles that provided (1) examples of how these tools are used and (2) insights concerning the strengths and limits of these methodological approaches in relation to the above three analytical criteria. 

In a second step, we searched the scientific literature to identify additional studies, reviews, and methodological articles that either used emerging statistical tools or discussed the strengths and limits of these tools in ways relevant to the above three analytical criteria. For this step, we searched the following databases: Scopus (including Medline) and Web of Science (including Science/Social Sciences/Emerging Sources and Arts and Humanities Citation Indexes). Many methodological developments relating to intersectionality have emerged from ideas and methodological roadblocks/advancements met in research using intersectional, embodiment, feminist, or ‘critical race theory’ frameworks [37]. Broad concepts, such as intersectional, embodiment, sex/gender, gender sensitive analysis, and race/racialization/ethnicity were searched and combined with the occupational health field. We also performed backward citation tracking to identify additional references. 

### 2.2. Screening and Selection

The inclusion criteria were the following:

- peer-reviewed article published in a scientific journal
- published between January 1980 and May 2022
- in French or English (the languages mastered by the authors)
- analyzing quantitative methods or using quantitative data analysis (excluding mixed or qualitative analytical tools)
- studies or reviews or methodological articles encompassing in-depth analyses of conventional (as defined in the first step of our search strategy) and/or emerging statistical tools
- allowing for the analysis of occupational health outcomes in diverse populations

Seventy-seven documents met the selection criteria and were included in our critical review.

### 2.3. Analytic Approach and Data Synthesis

The narrative synthesis consisted of mapping tools according to the type of statistical model applied (regression approaches, machine-learning approaches, and variance decomposition approaches) while distinguishing between commonly used conventional tools and newer emerging tools. We performed a critical analysis of the included articles using the following criteria:

(1) Contribution to identification of critical occupational determinants of health,
(2) Taking account of relevant population characteristics so as to reflect intersectional approaches to population health with a particular attention to sex/gender. The theoretical implications of the analysis regarding equity and potential dangers/adverse consequences of the results, such as stereotyping or increased stigmatization, were analyzed in detail,
(3) Feasibility: does the tool require sample sizes and population characteristics available to researchers for informative studies, since workplaces may employ relatively small numbers of women, minority workers, or older workers, for example? To give a broad order of magnitude, we consider a small sample size as being composed of ~30–100 participants, depending on the analysis [38]. Regression analyses will usually require a minimum of ~50 participants, with the number increasing the more independent variables are included (the rule of thumb being ~10 per independent variables added) and statistical analyses aimed at detecting differences between groups will usually require a minimum of ~30 participants per cell. Similarly, cluster analyses aim for sample sizes of ~20–30 per expected subgroup.

Each criterion was considered, and a data extraction was performed focusing on the strengths and limits of each statistical tool. The process of data extraction and analysis of the included articles was iterative. It led to the progressive refinement of our assessment. The results were integrated into a pragmatic methodological framework and organized around the discussion of the concerns emerging from the literature. This examination of each tool based on an in-depth analysis of the literature allowed us to identify the key elements that should be taken into account when choosing a statistical tool. These key elements are presented in Table 1.

## 3. Results

### 3.1. Conventional Models and Analytic Strategies

In this section, we analyze why the conventional fixed regression models with analytic strategies most commonly used in occupational health to account for sociodemographic variables-adjustment, stratification, and interaction testing fail to deal with the sex/gender-related and other sociodemographic phenomena that interact with occupational health and fail to grasp the interplay of these phenomena and their effects on employment and health equity with an intersectional lens. We will look at standard as well as multilevel regressions. 

A number of strategies will be examined, and our conclusions will be summarized in Table 1.

#### 3.1.1. Standard Regression Adjusting for Sex/Gender

In the past, sociodemographic variables such as sex/gender, age, class, immigration status, or ethnoracial identification have been treated as individual-level confounders in analyses relating occupational exposures to health effects [39,40]. That is, analysis of the “true” effect of an exposure is said to be “confused” by relationships between the confounder and both exposure and outcome. The adjustment strategy aims to cancel the effect of sex/gender, not to explore it, and this is a major limitation of this approach.

Another reason for not using this strategy is that the same exposure variable name may refer to different realities when applied to women and men. For example, “prolonged standing” among women workers more often involves static postures than among men, [9,41] “repetitive work” involves shorter repeat cycles for women [42,43], and “lifting weights” more often involves lifting people for women and lifting objects for men [44]. Static standing vs. moving around, fast vs. slow repetition, and lifting people vs. inanimate objects, have very different physiological effects and associations with health. By treating sex/gender as a confounding variable, information on unmeasured exposures that may be critical for prevention is lost, as when gender is a surrogate measure of exposure categories that have not yet been identified among potential causal factors [9,45]. This may occur even if interactions between sex/gender and other variables are tested [46].

Since self-reported sex/gender can be related to exposures and outcomes, it is important to have a method for relating exposures to outcomes that: (1) reveals possible male–female (sex) differences in reactions to the same exposure variables; (2) ensures that exposures of women and men (sex/gender) called by the same name correspond to the same physical and psychological stressors; (3) allows exploration of gender-modulated differences in exposure patterns so as to encourage prevention and compensation of occupational diseases in women’s occupations [47]; and (4) accords the same consideration to other relevant sociodemographic variables (such as ethnoracial identity, age, and disability) in order to improve accuracy in identifying harmful exposures while providing information useful for enhancing equity at work. These considerations were further analyzed during examination of the analytical methods considered below.

#### 3.1.2. Standard Regression with Stratification

An initial alternative to statistical adjustment is to stratify samples according to sex/gender [5]. Stratification can reveal exposure-effect relationships that are invisible when sex/gender is adjusted for [45]., It can also reveal deficiencies in sampling if women or men are proportionately less well represented. Additionally, when women and men are considered separately, scientists may think to include covariates or outcomes that only concern one sex, such as menstrual abnormalities and pain for women [48] and low sperm count for men [49]. 

However, stratification for sex/gender comes with its own set of limitations. A scientific problem comes from the fact that multiple other sociodemographic descriptors are associated with workplace exposures and outcomes, whether through discrimination (e.g., racism [50]), job and task segregation (e.g., immigrant status [51]), or physiological differences (e.g., age [52]). Some of them are continuous (e.g., physiology) or multicategorical (e.g., ethnoracial). Stratifying for all simultaneously is rarely possible with real-world sample sizes, given statistical power limitations

Even more importantly, stratification fails to fully engage with the complex ways in which these multiple identities, social positions and individual attributes interact and are embodied within different subgroups, affecting occupational risks and outcomes [19,53]. Similarly, several researchers have pointed out the danger of using validated instruments and analytic strategies based on a single sociodemographic marker or single axes of discrimination (e.g., race) and instead encourage researchers to use a ‘check all that apply’ approach in questionnaires and analyses (e.g., race, sex/gender, social class, and age) [54]. Researchers are encouraged to look beyond single sociodemographic markers to look at which characteristics may be present and relevant for the analysis, but also how this complex set of dimensions combine and contribute to both advantages and disadvantages towards the outcome, when analyzing and interpreting data [55,56,57]

Stratification may also carry the danger of contributing to sex/gender (or other) stereotyping. Even when sex/gender is not the principal characteristic of interest in the population being studied, stratifying may reinforce stereotypes if no explanation for the mechanisms underlying gender differences can be identified. For example, reporting that women working in traditionally male jobs have a much higher likelihood than men of having an occupational accident may encourage interpretations involving women’s “nature”, such as relative weakness or inability to handle machines, rather than resulting in pressure to adapt equipment and tools for a wider variety of human bodies [58].

#### 3.1.3. Additive and Multiplicative Approaches to Modelling Intersectionality

In response to the limitations mentioned above, researchers have called for methods able to better incorporate intersectionality in quantitative analyses [59]. To date, most of them have been conducted by examining categories of differences [18,53,60,61,62], often using either an *additive* or a *multiplicative* approach to regression [60].

##### Standard Regression with an Additive Approach

As with multiple stratification, standard regression with an additive approach assumes that sociodemographic variables have *additive effects* [18]. In other words, one of the statistical assumptions behind *additive approaches* to modelling intersectionality is that the sociodemographic variables are treated as completely independent from one another, so that occupational exposures and identity markers can be ranked and summed. For example, it assumes that, if we add the average effect of two categories of social positioning, such as being a woman and being racialized, we will obtain the cumulative effect of both identity markers (being a racialized woman). This is known not to be the case, as pointed out by Bowleg and Bauer [63], after analyzing a study by Schulman et al. [64] on the influence of age, gender, and race on referrals for cardiac catheterization. False patients who had different age/gender/racial characteristics were evaluated for referral. The additive approach led the authors to conclude that white people and men were more likely to be referred for this procedure than black people and women. However, in fact, intersectional analyses showed that, among black people and among women, only black women showed a lower level of referral. The use of the additive approach concealed the discrimination suffered only by black women.

##### Standard Regression with a Multiplicative Approach

*Multiplicative approaches* fit a model that includes additive “main effects” plus all possible combinations of sociodemographic categories as two-way, three-way, or more interaction terms [18]. In other words, this strategy isolates and adds up the contributions of independent variables (e.g., sex/gender, race, age, and occupation) to an outcome (e.g., work-related musculoskeletal disorders). It then corrects this artificial division of experience through the introduction of interaction terms, which operationalize the intersectionality at play [59]. In this kind of intersection-sensitive modelling, the additive model can be seen as a baseline on which occupational health researchers can, for example, apply the multiplicative approach to understand how women experience sexual and sexist harassment differently depending on their position in the work hierarchy, their immigration status, their education, or their sexual minority status [61,62,65].

However, these approaches also have their limits, since the interactions continue to be interpreted with regard to the main effects, for example, the effect of the sex/repetitive movements interaction will be interpreted in regard to the effect of sex alone or exposure to repetitive movements alone. It does not necessarily make sense to do this if we fully embrace the holistic embodiment or intersectionality frameworks. McCall [53] argues that this treatment erroneously assumes that the individual components of these interactions (e.g., being female or racialized) have a social meaning on their own. Statistically, however, the main effects must be included in the regression model when adding an interaction to avoid the risk of statistical misclassification [55].

Sample size limitation is another challenge to the *multiplicative approach* to testing interactions when doing multiple regressions. Many datasets are large enough to handle testing statistical interactions among a few pairs of variables. However, testing a greater number of interactions or higher-level interactions among three variables or more requires a lot more statistical power, as well as the presence in the sample of sufficient numbers of people belonging to the various social groups and their combinations. Both of these pre-conditions are rarely met in occupational health research, which often relies on small or medium-sized datasets collected in specific workplaces. Veenstra suggests that this limitation can in part be addressed in a technical way by using a higher *p*-value cut-off, such as *p* < 0.10 instead of *p* < 0.05 [62]. However, it does not address the other limitation stated above, which is that the interactions continue to be interpreted with regard to the main effects.

The exploration and interpretation of two-way or higher-order interactions can also be difficult and require a strong theoretical and experiential grounding in both the causal pathways and the intersectional dynamics that may be at play [59,66]. Several authors [28,56,57,59,61] describe these interpretive challenges as potential issues severely limiting insights into intersectionality. They consider that clarifying the theoretical grounds in which the analysis will take place is a critical pre-analysis step that should occur prior data collection. They also stress the importance of interpreting data within their socio-historical context, which requires collecting such contextual data [59]. In standard regression with a multiplicative approach, contextual factors are introduced in the models as individual-level variables and then included in higher-order interaction terms in order to assess their impact [59]. The fact that the collective context (e.g., membership in a small minority in a given workplace) is not considered as a level of its own in the analysis also limits the consideration of multiple social forces, factors, and power structures as variables interacting at several levels (organizational and societal) to shape and influence individual occupational outcomes [18,65].

#### 3.1.4. Multilevel Regression Modelling to Include Broad Social Forces

In order to reconcile standard multiple regression with contemporary approaches, researchers considering sex/gender and occupational health have called for methodologies allowing consideration of the complex intertwined levels at which sex/gender operates [67,68,69,70]. These levels can include the micro level of lived experiences, the meso level of labor division or organizations [71], and the macro level of social structures or sociohistorical contexts in which oppression or privileges develop [53,72], including internationally [73]. 

Multilevel regressions can model data measured at the individual level as well as data collected at higher levels, such as organizational (occupations, departments, or workplaces), characteristics of work disability compensation systems varying from province to province, etc. Meso factors can also be included among individual descriptors in conventional regression models, but the latter are limited to addressing the nested nature of individual versus meso versus societal characteristics and interactions between levels [70]. Multilevel models recognize the existence of such data hierarchies by allowing for residual components at each level. For example, in a two-level model, the residual variance could be partitioned into a between-workplaces component (the variance of the workplace-level residuals) and a within-workplaces component (the variance of the worker-level residuals). The workplace-level residuals, which one could call ‘workplace effects’, represent unobserved workplace characteristics that affect outcomes. Attention to these unobserved variables could lead to discoveries of similarities and differences among outcomes for workers from the same workplace. Therefore, the multilevel analysis could potentially identify workplaces with particularly toxic exposures, as well as individual variations in exposure or susceptibility, within a single model. Such analyses would allow researchers to examine hypotheses concerning the effects of sexist or racist practices on the relationship between repetitive work and absence for musculoskeletal disorders by departments, industries, or provinces.

Multilevel models can also be fitted to non-hierarchical structures. For instance, workers might be nested within a cross-classification of both workplaces and occupations in order to consider, in one single model, interindividual variations, variations among workplaces (grouping workers by employers, regardless of their occupations or job tasks), and variations among occupations (grouping workers from various employers into homogeneous occupations or job tasks). In such a non-hierarchical multilevel model, occupation factors and workplace factors are not considered to be nested, but both types are portrayed as operating at a higher level than the individual characteristics.

One of the advantages of multilevel models is also a limitation in that they require access to data collected at meso- or macro-levels. Large population health databases and worker compensation databases that are usually used in occupational epidemiology contain data almost exclusively collected at the individual level. When used to investigate intersectional effects, conventional multilevel models run into the same limitations as conventional fixed-effect regressions in terms of sample size requirements and the number of interactions they can accommodate in order to account for multiple categories of social identities. These limitations make it difficult to give the same consideration to several relevant sociodemographic variables simultaneously so as to improve accuracy in identifying harmful exposures while also providing information useful for enhancing equity at work.

### 3.2. Emerging Quantitative Intersectional Approaches

Several emerging statistical tools have been developed in the literature in the recent years, addressing some of the limitations stated above and major headway continues to be made on these innovative approaches. They offer promising avenues for conducting intersectional studies in occupational health.

#### 3.2.1. MAIHDA

Multi-level analysis of individual heterogeneity in discriminatory accuracy (MAIHDA) is a two-level model based on individual data [74,75]. Social positions are entered in the model at the first level and interactions or intersectional social strata at the second. By using multilevel analysis to model health inequalities within and between strata defined by the intersection of gender with multiple occupational, social, and demographic dimensions (for example), these models could provide a better understanding of the health heterogeneity existing in the worker population [75]. This inductive methodology allows for gathering data on many stratum-specific interactions of effects and simultaneously informs on the discriminatory accuracy of such strata for explaining or predicting individual health outcomes. It provides an answer to the call for quantitative methodologies well adapted to explore a greater number of interactions, within the intersectionality framework [76]. Another advantage of the MAIHDA method is that it remains valid even when testing many intersections (>100) [74,76]. Still another advantage is that it can be used with data collected at the individual level only, permitting the use of large administrative databases, clinical records, or survey data and the use of cross-sectional data (collected at one point in time). Identities and other indicators of social positions are here used as proxies for sets of social experience within interlocking power structures and systems of marginalization [75]—not necessarily as individual-level determinants of the occupational outcome. MAIHDA models help investigate risk factors while also avoiding the ‘tyranny of the averages’ [74,77]. They allow concurrent investigation of variations ‘between’ and ‘within’ populations, and increase discriminatory accuracy, recognizing the inability of ‘subpopulations’ or ‘strata’ to discriminate alone between those who will develop occupational diseases and those who will not.

#### 3.2.2. Decision Tree Methods

These methods, also called classification tree methods, are used for descriptive data analysis, and include C-Tree, CHAID, and Random forest. They mobilize artificial intelligence [78] to explore combinations of identity, status, occupational exposures, and produce outcome estimates for these intersections.

In simple classification tree methods (C-Tree, CHAID), researchers identify variables or categories of importance to enter in the algorithm. The analysis starts from the complete sample under investigation and successively splits the sample according to certain criteria (gender/sex, age, ethnicity, etc.) until splitting is no longer relevant because groups that meet a ‘stopping criteria’ at a certain ‘node’ in the tree can be interpreted as groups sharing a homogenous outcome. For such groups, further splitting would just lead to more groups with the same outcome. This type of analysis leads to a visual representation in the form of a descriptive tree identifying and characterizing homogeneous groups in terms of outcome, as well as their shared characteristics. A third classification tree method, called CART analysis, has often been used in quantitative intersectional analyses. However, Mahendran [79] showed recently that CART analyses tended to perform poorly in terms of producing non-biased estimates) in the context of descriptive intersectional analyses. So, when selecting a simple decision tree method for sex/gender or more general intersectional analyses, CTree or CHAID should be favored over CART.

The Random forest method is slightly different, as it uses bootstrap techniques to aggregate between various decision trees, hence the ‘forest’ [79,80]. Bootstrap sampling involves drawing sample data repeatedly with replacement from a data source to estimate a population parameter. In aggregating multiple decision trees formed from bootstrapped samples, the random forest is less susceptible to overfitting [80] (i.e., producing an analysis that corresponds so closely or exactly to a particular set of data that it becomes meaningless). This method leads to a variable importance measure (VIM), rather than a decision tree. VIMs are useful for identifying the most important variables for explanatory or predictive purposes. They may lead to the discovery of new risk or protective factors involved in a particular mechanism.

These methods have, for example, been used to predict low back pain among hospital staff using various individual and occupational factors (e.g., standing, sitting, body mass index, domestic activity level, child care, age, and marital status) [81] or interactions among disability, gender, age, ethnoracial identification, and employer characteristics when considering the proportion of harassment versus other forms of discrimination allegations [82]. This type of analysis widens the range approaches available to prevent low back pain, while including intersectional considerations.

As with the MAIHDA method, one of the advantages of decision tree methods is that they are not limited in the number of interactions that can be tested. Recent work from Mahendran et al. [79] on classification tree methods showed that CTree, CHAID, and Random forest methods all performed better than conventional main effects regression for the purpose of intersectional descriptive analyses, with both small and large sample sizes. However, when dealing with small sample sizes, MAIHDA should still be preferred, as decision tree methods require a moderate to large sample to obtain similar statistical power [79].

#### 3.2.3. Cluster and Latent Analysis

When examining causal pathways, as opposed to describing inequalities, even the most sophisticated decision tree techniques become limited. Latent variable or clustering methods can incorporate sex/gender in a more holistic way by considering individuals as the embodiment of their specific situation in regard to the exposure–outcome relationships at issue [20,26]. Bodies tell stories about the conditions in which they live, whether or not these are consciously recognized or expressed, and they “embody” multiple identities or social positions in a blend whose elements cannot be studied separately [83]. 

Using these methods, variables relevant to exposures can be used to map individuals to a many-dimensional space. Those with similar experiences will form clusters that can then be examined for the presence of variables of interest other than those used to form the clusters. For example, if similarities in working conditions are used to form the clusters, health outcomes, industries, and sociodemographic descriptors can be mapped onto the clusters.

Cluster and latent analyses can help reveal systemic effects. They can, for example, reveal gender differences in health behaviors, seeking or accessing care, exposure to and effects of occupational risks, as well as patterns in sexual division of labor and processes affecting men and women differently, e.g., harassment [84].

More generally, these statistical techniques can identify unmeasured subgroups (latent classes) based on individuals’ similarities in regard to observed variables. Latent class analysis (LCA) uses *categorical* variables (for example, derived from multiple choice questions) to identify these latent classes. Latent profile analyses (LPA) use similar techniques to create subgroups, such as using *continuous* variables (for example, exposures measured on a continuous scale), while Latent transition analyses (LTA) are used for longitudinal data (such as work disability duration).

Another family of methods includes analytical approaches such as hierarchical cluster analysis (HCA) and non-hierarchical cluster analysis (e.g., K-means clustering) [20,21]. In hierarchical cluster analyses, each observation in the dataset starts as its own cluster and is merged with similar clusters; pairs of clusters are then merged and move on to the hierarchy (agglomerative method). A top-down approach is also possible, in which all observations start in one cluster, and splits are performed over and over, as one moves down the hierarchy (divisive method). The goal is data-driven identification of clusters which, again, are uniform in regard to the attributes used to form them, but heterogeneous in regard to other identified clusters or constellations of attributes.

These techniques can be used in descriptive or exploratory analyses, but can also represent the first step in an analytical modelling in which clusters are subsequently used to predict outcomes in regression analyses, structural equation modelling, or survival analyses [20]. In these analyses, clusters identified with data measured at baseline are used in conjunction with follow-up data to study how belonging to a certain class or cluster is associated with later outcomes. 

The identification of these classes in relation to subsequent outcomes can lead to a fuller understanding of how sex and gender affect health in relation to other sociodemographic characteristics or identities, showing clearer patterns and systemic effects not otherwise captured. These techniques can allow researchers to use multiple sex and gender indicators across varying socioecological levels to arrive at nuanced understanding of the association of sex/gender and health. This procedure is different from intercorrelation-based understanding [85], where each variable’s interactions are tested separately. This kind of analysis enables a different understanding of sex and gender dynamics and patterning by including a variety of sex indicators (sex assigned at birth, but also, if available, sexual characteristics through biomedical measures such as chromosomes or hormone levels), as well as gender indicators (self-reported gender identity, behaviors/expression/personality traits, etc.) and by identifying patterns existing in the data, in an inductive way.

#### 3.2.4. Structural Analyses and Variance Decomposition Approaches

Two other groups of approaches are worth discussing: *path analysis/structural equation modeling*, and *three-way causal mediation decomposition* [86]. Researchers here consider whether or not there are differences in potential causal relationships across various social position characteristics (e.g., sex/gender, age, and ethnoracial identification), looked at across specific intersections, chosen based on academic studies, experience, accessing community knowledge, or on hypotheses as to why they are important potential mediators. Possible effect modification is then tested across these intersections. These analyses are based on the premise that a specific working condition could have a different weight for a specific group at a specific intersection and in specific social and historical contexts. Bauer [86] gives the example that being called names potentially affects an upper-class white man differently from a precarious racialized woman with a history of oppression.

In *path analysis and structural equation modeling* [87], the influence of the moderating variable is assessed on a hypothesized mediated relationship between the main exposure and a health outcome of interest (potentially important mediating intersection). The test evaluates whether the indirect effect of a mediation analysis is modified by different levels of another variable. If the mediation analysis (first step) confirms an indirect pathway, then the moderation hypothesis (second step) is tested. Structural equation modeling can also be used to define latent constructs within the observed data.

In the *three-way causal mediation decomposition*, Bauer, et al. [86] adapted Vanderwheels’ decomposition of inequality measures [88] so as to allow for the assessment of exposure-mediator interaction and define direct and indirect effects within the counterfactual framework (i.e., the situation in which the mediator, for example gender-based discrimination, would be absent). The authors describe actual and adjusted intersectional inequalities in psychological distress and decompose them to identify the expected inequality in outcome for various intersectional groups in three ways:

(1) effects due to unequal levels of discrimination; (2) effects due to membership in the more discriminated-against group that would have happened if its members had experienced the same lower levels of discrimination as the reference intersection; (3) effects due to unequal effects of the same levels of discrimination in different individuals.

In other words, the three-way decomposition allows researchers to answer the following questions: (1) what is the effect of experiencing different levels of discrimination? (2) If all groups were exposed to a low level of discrimination, what residual inequality would be expected to remain? (3) Do identical levels of discrimination have identical effects on a health outcome for all groups (or does the same level of discrimination affect psychological distress differently for different groups depending on their sociohistorical power struggles, characteristics, etc.).

Table 1 summarizes the major differences among the statistical tests, as well as their potential applications.

## 4. Discussion

### 4.1. Main Findings

We have examined several techniques for quantitative analysis in studies of occupational health in diverse working populations. Our goal was to suggest ways that such analyses could give a more accurate portrait of exposure-effect relations and enhance workplace equity as well as contributing to occupational health. Our examination of a selection of analytical techniques led us to conclude that: 

Adjusting and stratifying for sex and gender is not appropriate for analyses intended to integrate multiple relevant population characteristics in a way that takes advantage of intersectional approaches to population health and accurately reveals exposure-effect relationships. Demographic descriptors, such as gender, age, ethnoracial identification, or socioeconomic status, have historically been treated as ‘confounders’ in standard multivariable regression models, in order to ‘neutralize’ (and thereby make invisible) their effects on causal relationships between occupational exposures and health outcomes. When the disadvantages of adjustment for confounders were recognized, researchers called for stratified analyses. These have the advantage of making the descriptors and their effects visible, but with the concomitant risks of requiring large sample sizes, of encouraging stereotyping, and of misrepresenting groups at the intersections of identities. In particular, as Cisneros [37] puts it, *“intersectionality captures* ‘*the failure of feminism to interrogate* race’ *and* ‘*the failure of antiracism to interrogate patriarchy*’”. Understanding these systemic issues requires going beyond silo approaches to arrive at an integrated understanding of the determinants of health. We must also recognize that these demographic descriptors carry a heavy baggage of discrimination and even oppression and need to be treated carefully. 

Emerging quantitative approaches, such as MAIHDA, cluster and latent analyses, structural analyses, and mediation approaches, offer the possibility of mobilizing large data sets, such as work disability compensation databases, and address previous limitations to the number of interactions that can be examined at once and to the ways these intersections can be managed. Specifically, the MAIHDA model, as a multilevel regression application, evaluates interaction effects in fundamentally different ways from standard multilevel regression models. It is effective and powerful when it comes to identifying strata and handling large numbers of intersections while integrating an ecological perspective [75,89]. Clustering methods and latent analyses have not only been used to identify systemic patterns and render inequalities visible, but they have also been used, as a first step, in causal inferences, for example, as a way to create process-related classes of experiencing discrimination [90].

These new methods provide clues to understanding the pathways linking gender, exposures, and health. They have the potential to identify vulnerabilities and discriminations or strengths and protective factors in clusters or subgroups that never caught the eye before [18]. 

### 4.2. Rethinking Definitions of Exposures, Outcomes, and Population Descriptors

During the present consideration of methods, we have underscored the importance of the way exposures, population descriptors, and outcomes are operationalized during data collection. Since the meaning of an exposure descriptor may vary from one demographic subgroup to another, it is particularly important to give precise definitions to exposure variables. As an example, “standing at work», a category of interest in a number of research efforts concerned with cardiovascular symptoms [91,92], or low back pain [93], are often ill-defined [94]. Definitions vary from “*postures other than sitting*” [95] to *moving within a radius of less than one meter*/*less than 5 meters*/*over 5 meters* [96]. When the standing variable is ill-defined, researchers can detect a spurious relation between gender and health effects of standing, attributable in fact to a relation between gender and type of standing posture [9]. Analogous reasoning applies to variables, such as “repetitive work” (because women’s repetitive work tends to be high-repetition, low-force compared to men’s which is more often low-repetition, high-force [42]) or “variable work schedule” (because women’s and men’s family responsibilities tend to interact differently with variable work schedules [97]). Additionally, since women’s and men’s metabolism of some environmental toxins may differ and relatively little is known about women’s metabolism of toxins [98], exposure categories may have been designed inappropriately. Analogous arguments can apply to studies with, for example, racialized populations, to the extent that they are differentially integrated into professions, workplaces, and society at large.

Outcome measures can be subject to analogous bias. During the COVID-19 pandemic, health care workers became aware that people with darker skin were being under-diagnosed with occult hypoxemia due to the measuring devices used [99]. Other studies have shown gender or ethnic biases in clinical diagnoses, with symptom reports from some populations being minimized or treated with skepticism [100]. Bias in research funding and data collection have also been reported. Some outcomes are less studied than others and have received very little attention in relation to occupational exposures. Examples are dysmenorrhea [101], urinary incontinence [102], and workplace-related permanent health alteration among pregnant women (as opposed to fetuses) [103,104,105,106].

Finally, populations themselves can be difficult to describe. Defining sex and gender is increasingly complicated [107]. Depending on the postulated underlying gendered and biological pathways to health, a combination of gender and sex indicators are needed to operationalize these concepts. Defining and identifying populations in terms of their likelihood of undergoing discrimination can be tricky as well [108].

### 4.3. Studying Disadvantaged Populations without Creating Further Sources of Stigmatization or Discrimination

Identifying and providing information on certain disadvantaged or more at-risk groups can, if conducted without precautions, lead to the stigmatization of these groups or even increase their exclusion or the discrimination to which they are subjected. This is why some public health researchers in Quebec objected to the collection of ‘race-based data’ during the COVID-19 pandemic, although other provinces, such as Ontario, did gather such data [109]. Even if these data are seen as essential for the protection of groups at risk and for correcting occupational health inequalities, the fear of seeing them interpreted in a way that reinforces stigma and discrimination is not unfounded. Extreme care must be taken concerning the way such results are communicated, especially to protect against discrimination in employment.

One of the ways to reduce or mitigate the risk of encouraging stereotyping and discrimination is to go beyond the simple quantification of inequalities to analyze and contextualize the results. This involves not only nuanced operational definition of variables, but also collecting data on the context surrounding the research situation and reporting on them, along with the other results.

It is also important to report the results accompanied by potential explanations of the phenomena observed. What mechanisms, what institutional contexts and what social processes are at play that make it possible to understand the differences in exposures and/or effects observed? Bowleg [54] talks about interpreting results through a sociohistorical perspective on social inequities to understand how certain identities and circumstances impact health. Chappert [110] refers to the necessity of ‘*putting on gender glasses*’ to understand and correctly interpret the variations between men and women in terms of exposures and health effects at work. In all cases, it is a question of delivering and interpreting the results while being aware that the effects that crystallize around socio-demographic characteristics often result from power imbalances and social inequities and reflect policies and practices that maintain them [17]. By focusing on systemic workplace effects and the policies, power structures, regulations, and institutions that enable or sustain them, rather than on individual factors, one is more likely to uncover realistic, sustainable, and achievable ways to remedy both the health problems and the injustices that are identified.

### 4.4. The Role of Participatory Research Approaches in Favoring Equity in the Workplace

If the aim of intersectional analysis is to prevent occupational illness in diverse populations, it has been suggested that this can only be done by involving members of discriminated groups to orient and inform such analyses and interpretation [39,111]. Such endeavours are not easy. Quantitative analyses are by their nature technical and not readily accessible, so researchers need to learn how to explain them. There are many moments throughout the research process, from research question conceptualization and data collection to explanations and interpretations, during which validation can be sought with populations concerned, and these exchanges can provide exciting new paths for exploration. Participatory approaches involving diverse representatives of workers and employers are therefore recommended.

### 4.5. Limitations

Introducing intersectionality and embodiment frameworks into quantitative approaches comes with great potential to contribute to the analysis of occupational health issues and, ultimately, to improve health equity. However, some loss occurs when trying to reconcile the complexity of these theories and their core tenets with the operational needs and conditions necessary for statistical modelling.

On a pragmatic level, quantitative methods such as regression require researchers to ‘put people in boxes’ because they generate variables using categorizations according to various attributes. It can be argued that categorization irremediably oversimplifies any attempt at quantitative intersectional analyses. This is because, unlike qualitative methods, it cannot capture individual and subjective lived experience within its sociocultural and historical context, communities, and power relations [19]. However, the power of quantitative studies to reveal problems and stimulate change is undisputed, and categorization is therefore unavoidable. It is still possible to question the assumption that categories are fixed [28]. For the tenants of the *anti-categorical complexity approach*, such as that taken by post-structuralist and deconstructivist feminists [112], this assumption is irreconcilable with the intersectional paradigm that considers social positions as a product of dynamic power relations that are in constant redefinition. In this view, categories are the result of linguistic processes and, as such, should be rejected to concentrate on inclusion and exclusion mechanisms [113] through the use of qualitative methodologies. We and others [53] instead see categorization as a necessary compromise that needs to be made if one wants to benefit from the potential that quantitative data have to offer.

In this critical review exercise, we have not examined all possible statistical strategies, but have selected those we felt to be most promising. We recognize that many available data bases will not contain all the information necessary to deploy all of these tools in an optimal way.

Although our concern with the equitable treatment of sex and gender was at the heart of the present exploration, we have not discussed in detail the meaning of sex and gender, nor gone deeply into the complexities of definition of these entities [107,114,115].

## 5. Conclusions

We have found that the statistical techniques currently used to take account of population characteristics in occupational epidemiology (adjustment and stratification) present some deficiencies when relating exposures to health effects. In contrast, intersectional and embodiment frameworks carry great potential to improve our understanding of occupational health inequalities and should continue to be encouraged in occupational health research. Although the integration of these frameworks in quantitative research is imperfect and poses complex methodological challenges, it can provide powerful insights into situations in which social positions interact with occupational and non-occupational health risks and exposures within interlocking systems of privilege and oppression. Our consideration of several methods suggested by applying these frameworks concludes that quantitative analyses using them may be useful in attempts to improve occupational health.

## Figures and Tables

**Table 1:** Characteristics of included statistical tools for identifying exposure-effect relations with an intersectional lens.

	Method	Accurate Estimation with Small Sample Size?	Accommodates Many Intersections?	Data-Driven or Theory Driven?	Multilevel?	Effect Size Estimates?	This Analysis Is Recommended for
Regression approaches	Standard regression with adjustment strategy	Yes	No	Theory driven	-	Yes	Not recommended
	Standard regression with stratification strategy	No	No	Theory driven (Subgroups chosen using available information)	-	Yes	Large sample sizes with limited number of intersections (not a lot of subgroups), when researchers have a good understanding of underlying mechanisms (to avoid the risk of stereotyping).
	Standard regression with additive approach to intersectionality	Yes	No	Theory driven	-	Yes	Not recommended
	Standard regression with multiplicative approach to intersectionality (through interactions testing)	No	No	Theory driven	-	Yes	Not recommended
	Multilevel regression	No	No	Theory driven	Yes	Yes	Research designs where data for participants are organized at more than one level (nested data), when researchers are aware of limitations to standard regression approaches
	MAIHDA	Yes	Yes	Theory driven	Yes	Yes	Estimating effect sizes in samples of varying size with large numbers of intersections and in research designs where data for participants are nested in their intersectional strata
Machine learning approaches	Decision trees	No	Yes	Data driven (the subgroups emerge from the data analysis)	-	No	Identification of subgroups and detection of variable combinations relevant to outcome (combination of exposures, sociodemographic identifiers, social determinants, etc.)
	Cluster analysis	Yes	Yes	Data driven	-	No	Identification of subgroups and detection of variable combinations relevant to outcome (combination of exposures, sociodemographic identifiers, social determinants, etc.)
	Latent class analysis	Yes	Yes	Data driven	-	Yes	Identification of subgroups and detection of variable combinations relevant to outcome (combination of exposures, sociodemographic identifiers, social determinants, etc.)
Variance decomposition approach	SEM/path analyses	Yes	Yes	Theory driven	-	Yes	Evaluation of the influence of potentially important mediating variable between the main exposure and a health outcome of interest or definition of latent constructs within the observed data
	Three-way causal mediation decomposition	Yes	No	Theory driven	-	Yes	Identification of the expected inequality in outcome for various intersectional groups, in three ways: (1) residual inequality of effect if all groups were exposed, (2) effect of experiencing different levels of discrimination resulting in exposure differences, and (3) effect of identical levels of discrimination on effects in different groups

## Data Availability

Not applicable.
